# Chlorhexidine *versus* povidone–iodine skin antisepsis before upper limb surgery (CIPHUR): an international multicentre prospective cohort study

**DOI:** 10.1093/bjsopen/zrab117

**Published:** 2021-12-15

**Authors:** Ryckie G Wade, Gráinne Bourke, Justin C R Wormald, Joshua Philip Totty, Guy Henry Morton Stanley, Andrew Lewandowski, Sandeep Singh Rakhra, Matthew D Gardiner, The CIPHUR Collaborative, R Bindra, M Sher, M Thomas, S D J Morgan, B Hwang, W Santucci, P Tran, L Kopp, V Kunc, A Hamdi, P P Grieve, S A Mukhaizeem, K Blake, C Cuggy, R Dolan, E Downes, E Geary, A Ghadge, P Gorman, M Jonson, N Jumper, S Kelly, L Leddy, M E McMahon, C McNamee, P Miller, B Murphy, L O'Halloran, K O’Shea, J Skeens, S Staunton, F Timon, J Woods, U Cortinovis, L Sala, V Zingarello, M H Jusoh, A N Sadagatullah, G Georgieva, S Pejkova, B Nikolovska, B Srbov, H K S Hamid, M Mustafa, M Abdelrahman, S M M Amin, D Bhatti, K M A Rahman, I Jumabhoy, J Kiely, I Kieran, A C Q Lo, K Y Wong, A Y Allan, H Armes, M D Horwitz, L Ioannidi, G Masterton, H Chu, G D Talawadekar, K S Tong, M Chan, M Tredgett, C Hardie, E Powell-Smith, N Gilham, M Prokopenko, R Ahmad, J Davies, S Zhen, D Dargan, R M Pinder, M Koziara, R Martin, E Reay, E Cochrane, A Elbatawy, F Green, T Griffiths, G Higginbotham, S Louette, G McCauley, I Natalwala, E Salt, R Ahmed, P Goon, R Manton, N Segaren, G Cheung, R Mahoney, S Sen, D Clarkson, M Collins, A Bolt, P Lokanathan, A Ng, G Jones, J W M Jones, R Kabariti, S J Rhee, J Herron, A Kay, L K Cheung, D Thomson, R S Jugdey, H Yoon, Z L, J Southgate, C Brennan, S Kiani, M Zabaglo, Z A Haider, R Poulter, A Sheik-Ali, A Watts, B Jemec, N Redgrave, L Dupley, M Greenhalgh, J Vella, H Harris, A V Robinson, S Dupre, S Teelucksingh, A Gargan, S Hettiaratchy, A Jain, R Kwasnicki, A Lee, M Thakkar, D Berwick, N Ismail, M Mahdi, J Rodrigues, C Liew, A Saadya, M Clarkson, C Brady, R Harrison, A Rayner, G Nolan, B Phillips, N Madhusudan

**Affiliations:** Leeds Institute for Medical Research, University of Leeds, Leeds, UK; Department of Plastic and Reconstructive Surgery, Leeds Teaching Hospitals Trust, Leeds, UK; Leeds Institute for Medical Research, University of Leeds, Leeds, UK; Department of Plastic and Reconstructive Surgery, Leeds Teaching Hospitals Trust, Leeds, UK; Nuffield Department of Orthopaedics, Rheumatology and Musculoskeletal Sciences, University of Oxford, Oxford, UK; Department of Plastic and Reconstructive Surgery, Hull University Teaching Hospitals, Castle Hill Hospital, Cottingham, East Riding of Yorkshire, UK; Centre for Clinical Sciences, Hull York Medical School, Hull, UK; Department of Plastic Surgery, Fiona Stanley Hospital, Murdoch, Western Australia; Department of Plastic Surgery, Mater Adults Hospital, Raymond Terrace, South Brisbane, Queensland, Australia; Department of Anaesthesiology, Alfred Health, Melbourne, Victoria, Australia; Nuffield Department of Orthopaedics, Rheumatology and Musculoskeletal Sciences, University of Oxford, Oxford, UK; Department of Plastic Surgery, Frimley Health NHS Foundation Trust, Slough, UK

## Abstract

**Introduction:**

Surgical site infection (SSI) is the most common and costly complication of surgery. International guidelines recommend topical alcoholic chlorhexidine (CHX) before surgery. However, upper limb surgeons continue to use other antiseptics, citing a lack of applicable evidence, and concerns related to open wounds and tourniquets. This study aimed to evaluate the safety and effectiveness of different topical antiseptics before upper limb surgery.

**Methods:**

This international multicentre prospective cohort study recruited consecutive adults and children who underwent surgery distal to the shoulder joint. The intervention was use of CHX or povidone–iodine (PVI) antiseptics in either aqueous or alcoholic form. The primary outcome was SSI within 90 days. Mixed-effects time-to-event models were used to estimate the risk (hazard ratio (HR)) of SSI for patients undergoing elective and emergency upper limb surgery.

**Results:**

A total of 2454 patients were included. The overall risk of SSI was 3.5 per cent. For elective upper limb surgery (1018 patients), alcoholic CHX appeared to be the most effective antiseptic, reducing the risk of SSI by 70 per cent (adjusted HR 0.30, 95 per cent c.i. 0.11 to 0.84), when compared with aqueous PVI. Concerning emergency upper limb surgery (1436 patients), aqueous PVI appeared to be the least effective antiseptic for preventing SSI; however, there was uncertainty in the estimates. No adverse events were reported.

**Conclusion:**

The findings align with the global evidence base and international guidance, suggesting that alcoholic CHX should be used for skin antisepsis before clean (elective upper limb) surgery. For emergency (contaminated or dirty) upper limb surgery, the findings of this study were unclear and contradict the available evidence, concluding that further research is necessary.

## Teaser

This international study recruited 2454 children and adults undergoing surgery on their upper limb (arm or hand), from 42 hospitals worldwide. The overall risk of infection after surgery was 3.5 per cent. Before planned (elective) surgery, if surgeons cleaned the skin with alcoholic chlorhexidine, then the risk of infection was reduced by 70 per cent.

## Introduction

Surgical site infection (SSI) is the most common and costly complication of surgery^1^^,^^2^, with broad-ranging ramifications for patients, healthcare systems and society. Potential consequences of SSI in the upper limb include: delayed return to work^3^, delayed rehabilitation which may reduce functional recovery^4^ and prevent independent living^3^, increased antibiotic consumption^5^, reoperation^6^, amputation^7^, and death from sepsis^8^.

To reduce the risk of SSI, the WHO^9^, the US Centers for Disease Control and Prevention (CDC)^10^, and the UK’s National Institute for Health and Care Excellence (NICE)^11^ recommend alcoholic chlorhexidine (CHX) for skin antisepsis. Alcoholic CHX has been shown to halve the risk of SSI following clean^12^ and contaminated^13^^,^^14^ surgery, when compared with other antiseptics such as povidone–iodine (PVI). However, upper limb surgeons continue to use other antiseptics, citing a lack of evidence pertaining to upper limb surgery^12^, as well as unresolved concerns over the safety of alcoholic CHX in the presence of open wounds and tourniquets^15–18^.

The global age-standardized incidence of injury to the upper limb exceeds 179 per 100 000 and despite improved health and safety standards, the incidence is not falling^19^. In the UK, the cost of hand and wrist injuries is estimated to be £460 million per annum^20^, which exceeds the cost of hip fractures (£335 million) and head injuries (£223 million). Equally, the number of patients being diagnosed with common hand conditions is increasing globally^21^^,^^22^ and consequently, the demand for elective upper limb surgery is rising^23^. Recent work suggests that over the coming decade, the demand for elective upper limb surgery will increase by 39 per cent^24^. Given the antimicrobial resistance crisis^25^, morbidity and mortality associated with SSI in the upper limb, and increasing rates of upper limb surgery worldwide, there is a pressing need to reduce SSI.

The aim of this study was to evaluate the current practice, safety and effectiveness of different topical antiseptics before upper limb surgery.

## Methods

This was an international multicentre, prospective cohort study of adults and children undergoing upper limb surgery. CIPHUR was advertised and collaborators were recruited via the UK’s Reconstructive Surgery Trials Network (RSTN), the Clinical Trials Network of Australia and New Zealand (CTANZ) and the Australasian Clinical Trials in Plastic, Reconstructive & Aesthetic Surgery (ACTPRAS) collaboratives. The study was registered at each participating hospital in accordance with local and national regulations. Informed consent was taken from patients if required by local or national regulations. In the UK, CIPHUR was registered as a service evaluation (so Health Research Authority (HRA) approval was not required as per the HRA decision tool) and collaborators were required to provide evidence of Caldicott Guardian approval before being registered. In Australia, the project was formally reviewed by the Townsville and Cairns Human Research Ethics Committees and defined as a quality assurance project, and thus exempt from ethical review. No changes were made to patients’ usual care in the conduct of CIPHUR. Routine, anonymized data were captured via the Research Electronic Data Capture (REDCap) web application^26^^,^^27^, hosted at the Kennedy Institute of Rheumatology, University of Oxford.

### Participants and procedures

Consecutive adults and children undergoing surgery (elective or emergency) distal to the shoulder joint were eligible. Patients with any active infection (anywhere in the body) at the time of surgery were excluded. Active infection was defined pragmatically as either a suspicion of infection or the provision of any medical or surgical treatment for either suspected or confirmed infection.

### Hospitals and settings

Any hospital offering upper limb surgery, in any location or setting, was eligible to participate. Collaborators were required to enrol consecutive patients (to mitigate selection biases) during the recruitment phase from 1st March 2020 to 31st December 2020 (see *Figure S1*).

### Outcome measures

The main outcome of interest was SSI. The WHO^9^, CDC^10^, and NICE^11^ define SSI as ‘infection within 30 days of an operation or up to 90 days if an implant is left in place’. However, there is no consensus on the diagnostic criteria for SSI; the available tools^28^ have poor agreement^29^, and defining explicit thresholds for clinical signs is impractical. Therefore, in this study, SSI was defined pragmatically as either clinically suspected or microbiologically confirmed infection, which required any form of medical and/or surgical treatment within 90 days of surgery. All patients enrolled within the study were subject to face-to-face or remote follow-up during the 90-day surveillance period, in accordance with local practices. Collaborators were not required to provide evidence for the criteria they used to reach a diagnosis of SSI. Other outcomes of interest included the occurrence of adverse events associated with antiseptic use, such as an ignition fire (with alcohol as the accelerant), a chemical burn beneath a tourniquet, or a hypersensitivity reaction.

### Variables used to model the risk of surgical site infection

Antiseptics were categorized into five groups: alcoholic or aqueous PVI or CHX and others (see *Table S1*). To make adjustments, data were also captured on variables associated with SSI in the upper limb, including: diabetes^30^; immunosuppression^9^; tobacco smoking^31^; and the CDC wound status (clean, clean-contaminated, contaminated, or dirty). Data were also collected on factors which remain contentious in hand surgery such as wound toilet at the time of assessment^32^, time from injury to surgery^33^, perioperative antibiotic use^34^^,^^35^, and materials used for wound closure^36^.

Wound toilet was defined as irrigation or bathing of the limb at the time of assessment. The solution used for wound toilet was collected in a mutually inclusive manner. Preoperative informal ‘social’ wash was defined as non-sterile cleaning of the upper limb before formal skin preparation. The grade of the operating surgeon was defined as follows: doctors within 4 years of graduation were defined as ‘junior trainees’; those who were 4 or more years postgraduate and in a plastics or orthopaedic specialty training post were defined as ‘specialist trainees’; and tenured specialists were defined as ‘consultants’. For the covariable wound closure, the ‘other’ category comprised patients who had their wounds closed using a combination of absorbable and non-absorbable sutures, those who healed by secondary intention, and those with no wounds to close (for example, percutaneous Kirschner wiring of a closed fracture).

### Missing data

Of the 2454 records submitted, 2294 (94 per cent) contained all data required to model the primary outcome and were thus considered to be 100 per cent complete. The primary outcome (SSI) was missing completely at random in 31 records (1 per cent). The overall rate of missing data was 4.1 per cent for the minimum data set required to model the primary outcome. Therefore, multiple imputations were not performed, and complete case analysis proceeded with^37^.

### Statistical analysis

The raw data and REDCap data dictionary are available via the Open Science Framework (https://osf.io/v6k8u/). Continuous variables which approximated the normal are presented as the arithmetic mean(s.d.) and compared using linear methods. Skewed continuous variables are summarized as the median with interquartile range (i.q.r.) and compared using the Mann–Whitney U test. Categorical variables are presented as frequencies with percentages and compared using the Fisher’s exact test.

To estimate the risk of SSI over time, mixed-effects time-to-event models (mestreg in Stata) were used for emergency and elective surgery patients separately. The categorical fixed-effects common-to-both models were antiseptic, diabetes, current tobacco smoking, wound toilet at assessment, preoperative antibiotics, grade of the operating surgeon, method of wound closure, and postoperative antibiotics. For emergency surgery, CDC wound status (level of contamination) and wound toilet were added as categorical covariables, and hours from injury to surgery as a continuous covariable. We planned to use ageas a covariable but decided to remove this factor, as the coefficient was effectively zero. The hospital (see *Figure S2*) was selected a random effect, and its variance was estimated using the Huber–White sandwich estimator. The Weibull distribution was used — in sensitivity analyses, Cox proportional-hazard models with shared frailty were also used and the results were essentially identical. Hazard ratios (HRs) and their 95 per cent confidence intervals are presented graphically^38^. Given that the date of SSI was missing in 31 records (1 per cent), further sensitivity analyses for the primary outcome were also performed (for elective and emergency surgery separately) using mixed-effects logistic regression. The same fixed and random effects were used, but the cluster-level variance was estimated via the restricted maximum likelihood method. The variances and covariances in all models were unstructured, and thus distinctly estimated. The term ‘statistical significance’ has been avoided^39^^,^^40^ and instead the focus is on clinical interpretation in relation to the point estimates and their respective 95 per cent confidence intervals.

### Estimating the cost of surgical site infection

The cost per SSI episode was estimated by use of additional care events recorded for patients experiencing infection. Over 90 per cent of costs related to infection are incurred as a result of additional inpatient stays^41^ and therefore, focus was placed on these costs. The estimated cost per infection was calculated from UK’s National Health Service (NHS) reference costs inflated to 2019/2020 prices^42^. A weighted average of two HRG codes (WH07C and WH07D) related to unplanned admission for surgical infection was used (see *Table S2*). Short admissions were defined as a stay of 2 days or less. For patients who did not require admission, it was assumed that at least one contact with primary care was made at a flat rate of £33.50^42^.

The effect of excess bed days, rather than readmission, upon cost per SSI was tested in a sensitivity analysis. A weighted average of the same two HRG codes related to excess bed days was used and inflated to 2019/2020 prices (see *Table S3*). An average cost per SSI was calculated by use of the mean additional inpatient stay due to SSI multiplied by the cost per additional bed day. This approach was expected to underestimate the cost per SSI (as it did not account for readmission costs or costs of visiting primary care) but was taken to indicate a reasonable minimum.

## Results

Overall, 2454 patients were included. Baseline demographics are presented in *Table 1*. Details of treatments provided to patients undergoing emergency and elective upper limb surgery are shown in *Tables S4* and *S5*. The overall risk of SSI was 3.5 per cent. Infection was more common following emergency surgery (57 of 1436 patients, 4.0 per cent), compared with elective surgery (28 of 1018 patients, 2.8 per cent). The median time to diagnosis of SSI was 15 (i.q.r. 7–28) days.

**Table 1:** Characteristics of the cohort

Characteristics		No SSI (*n* = 2338)	SSI (*n* = 85)
Mean (s.d.) age (years)		46 (21)	46 (20)
Sex	M	1480 (63.3)	60 (70.6)
	F	858 (36.7)	25 (29.4)
Risk factors for SSI	None	1778 (76.0)	55 (64.7)
	Diabetes mellitus	151 (6.5)	7 (8.2)
	Current smoker	254 (10.9)	15 (17.7)
	Immunosuppression	46 (2.0)	3 (3.5)
	Peripheral vascular disease	49 (2.1)	3 (3.5)
	Other risk factors	143 (6.1)	8 (9.4)
Urgency of surgery	Elective	973 (41.6)	28 (32.9)
	Emergency	1365 (58.4)	57 (67.1)
Type of surgical wound	Clean	1415 (60.5)	38 (44.7)
	Contaminated	846 (36.2)	42 (49.4)
	Dirty	77 (3.3)	5 (5.9)
Region operated on	Digits	1100 (47.1)	45 (52.9)
	Palm or dorsum	533 (22.8)	23 (27.1)
	Wrist	490 (21.0)	14 (16.5)
	Forearm	228 (9.8)	12 (14.1)
	Elbow	135 (5.8)	4 (4.7)
	Arm	127 (5.4)	7 (8.2)
Antiseptic	Aqueous povidone–iodine	602 (25.8)	34 (40.0)
	Alcoholic povidone–iodine	299 (12.8)	5 (5.9)
	Aqueous chlorhexidine	582 (24.9)	20 (23.5)
	Alcoholic chlorhexidine	772 (33.0)	22 (25.9)
	Others	79 (3.4)	4 (4.7)

Unless otherwise stated, values in parentheses are percentages. SSI, surgical site infection.

### Elective surgery

**Fig. 1:**
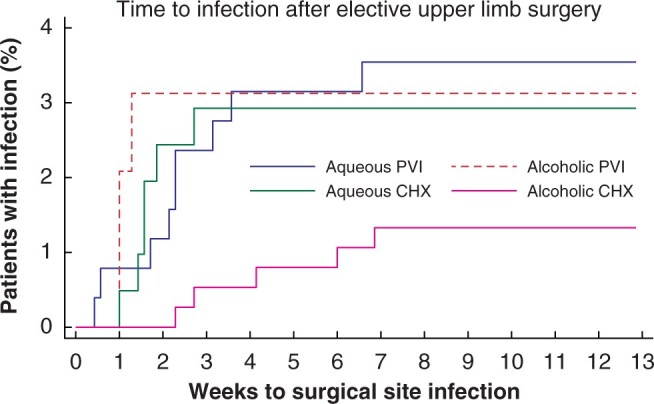
Kaplan–Meier plot of time to surgical site infection for different antiseptics in elective upper limb surgery PVI, povidone–iodine; CHX, chlorhexidine.

**Fig. 2:**
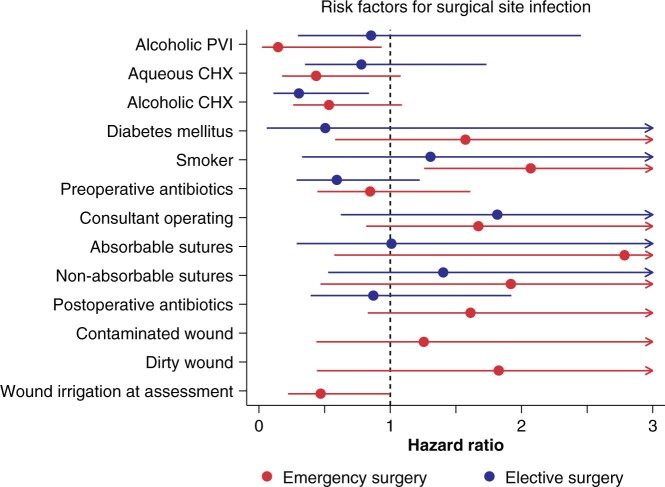
Forest plot showing the risk factors for surgical site infection PVI, povidone–iodine; CHX, chlorhexidine.

Alcoholic CHX was superior to all other antiseptics for elective surgery (1018 patients) (*Fig. 1*). The prevalence of SSI was 1.6 per cent for alcoholic CHX, 2.9 per cent for aqueous CHX, 3.1 per cent for alcoholic PVI, 3.5 per cent for aqueous PVI, and 5.80 per cent for other antiseptics. After adjustment, the time-to-event model suggests that if surgeons swapped from aqueous PVI to alcoholic CHX for elective surgery, then the risk of SSI might be reduced by 70 per cent (adjusted HR 0.30, 95 per cent c.i. 0.11 to 0.83) (*Table 2* and *Fig. 2*). The confidence interval around the estimate is wide, so the benefit might be as little as a 17 per cent reduction or as high as an 89 per cent reduction in the risk of SSI. The sensitivity analysis by use of mixed-effects logistic regression yielded similar findings (see *Fig. S3* and *Table S6*). Location (that is, the hospital) was not associated with any meaningful variability in the risk of SSI (see *Table S6*). Overall, the data indicate that alcoholic CHX was the most effective antiseptic for reducing SSI following elective upper limb surgery. If surgeons (who used other antiseptics) changed their practice and used alcoholic CHX, then the absolute risk reduction (ARR) would be 2.3 per cent, equating to a number needed to treat (NNT) of 44 to prevent one infection (see *Table S7*).

**Table 2:** Hazard ratios for surgical site infection derived from mixed-effects survival models

Risk factors	Unadjusted HR (95% c.i.)	Adjusted HR (95% c.i.)
**Emergency surgery**		
Aqueous povidone–iodine	Referent	Referent
Alcoholic povidone–iodine	0.15 (0.03, 0.79)	0.15 (0.02, 0.94)
Aqueous chlorhexidine	0.44 (0.20, 0.99)	0.44 (0.18, 1.08)
Alcoholic chlorhexidine	0.52 (0.27, 0.98)	0.53 (0.26, 1.09)
Clean wound	Referent	Rreferent
Contaminated wound	2.17 (0.89, 5.29)	1.26 (0.44, 3.60)
Dirty wound	3.13 (0.93, 10.6)	1.83 (0.44, 7.53)
Diabetes mellitus	1.57 (0.55, 4.52)	1.57 (0.58, 4.26)
Current smoker	2.18 (1.34, 3.55)	2.07 (1.26, 3.41)
Wound irrigation at assessment	0.45 (0.23, 0.86)	0.47 (0.22, 1.00)
Preoperative antibiotics	0.92 (0.50, 1.71)	0.85 (0.45, 1.61)
Consultant operating	1.28 (0.72, 2.24)	1.67 (0.82, 3.41)
Other wound closure method	Referent	Referent
Wound closure with absorbable sutures	2.79 (0.61, 12.9)	2.79 (0.57, 13.5)
Closed with non-absorbable sutures	2.47 (0.54, 11.3)	1.92 (0.47, 7.84)
Postoperative antibiotics	1.97 (0.99, 3.91)	1.61 (0.83, 3.13)
**Elective surgery**		
Aqueous povidone–iodine	Referent	Referent
Alcoholic povidone–iodine	0.88 (0.32, 2.46)	0.85 (0.30, 2.45)
Aqueous chlorhexidine	0.82 (0.34, 2.00)	0.78 (0.35, 1.73)
Alcoholic chlorhexidine	0.37 (0.15, 0.89)	0.30 (0.11, 0.83)
Diabetes mellitus	0.39 (0.05, 3.10)	0.52 (0.06, 4.24)
Current smoker	1.02 (0.25, 4.09)	1.31 (0.29, 5.27)
Preoperative antibiotics	1.00 (0.47, 2.14)	0.60 (0.29, 1.22)
Consultant operating	1.50 (0.55, 4.10)	1.81 (0.63, 5.27)
Other wound closure method	Referent	Referent
Wound closure with absorbable sutures	0.76 (0.20, 2.95)	1.01 (0.29, 3.53)
Closed with non-absorbable sutures	1.32 (0.41, 4.28)	1.40 (0.53, 3.73)
Postoperative antibiotics	0.84 (0.44, 1.60)	0.87 (0.39, 1.92)

HR, hazard ratio.

After adjustment, no other factors were associated with SSI (see *Table S4*). The use of antibiotics (before surgery or postoperatively) and the method of wound closure (absorbable *versus* non-absorbable sutures) were not associated with the risk of SSI.

### Emergency surgery

**Fig. 3:**
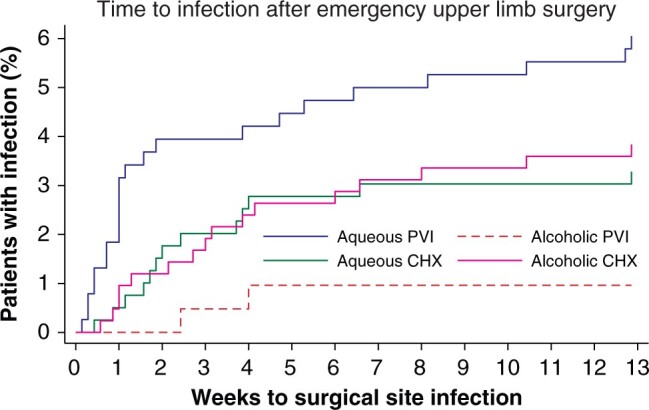
Kaplan–Meier plot of time to surgical site infection for different antiseptics in emergency upper limb surgery PVI, povidone–iodine; CHX, chlorhexidine.

The least effective antiseptic for preventing SSI in emergency upper limb surgery (1436 patients) was aqueous PVI (*Fig. 2*). After adjustment, CHX antiseptics in either aqueous or alcoholic form appeared to be better than aqueous PVI, and PVI in alcohol also appeared to be better than its aqueous counterpart (see *Table S4* and *Fig. 3*). The estimates indicate that if surgeons stopped using aqueous PVI and instead prepared the skin with alcoholic PVI, then the risk of SSI might be reduced by 85 per cent on average (adjusted HR 0.15, 95 per cent c.i. 0.02 to 0.94); however, the relative benefit may be as much as 98 per cent or as little as 6 per cent. Similarly, if surgeons stopped using aqueous PVI and switched to a CHX-based antiseptic (alcoholic or aqueous), then on average, the risk of SSI would be approximately halved (see *Table S4*). Sensitivity analyses by use of mixed-effects logistic regression (see *Fig. S3* and *Table S6*) also showed that aqueous PVI was the worst antiseptic for emergency surgery. It appeared that the risk of SSI varied substantially among hospitals (ICC 0.17, 95 per cent c.i. 0.04 to 0.50; 42 clusters), although this was attributable to one outlier. When this hospital was removed in a sensitivity analysis, the point estimates and confidence intervals were largely unchanged; however, the residual variance became effectively zero (ICC 1.49 × 10^−^^33^, 95 per cent c.i. 8.74 × 10^−34^ to 2.54 × 10^−33^; 41 clusters). This suggests that geography has little effect on the risk of SSI in emergency surgery. Overall, despite the uncertainty, the data suggest that aqueous PVI is the least effective antiseptic for preventing SSI following emergency upper limb surgery.

For emergency surgery only, after adjustment, smokers had more than twice the risk of SSI, compared with non-smokers (adjusted HR 2.07, 95 per cent c.i. 1.26 to 3.41) (*Table 2*). Similarly, wound irrigation at the time of assessment appeared to halve the risk of SSI (adjusted HR 0.47, 95 per cent c.i. 0.22 to 1.00) (*Table 2*). No other factors were associated with SSI, including the use of antibiotics (pre- or postoperatively) and the method of wound closure (absorbable *versus* non-absorbable sutures).

### Treatment of surgical site infection

The majority of patients with SSI were managed as outpatients (65 patients, 81 per cent), although an important minority (15 patients, 19 per cent) were readmitted for a median of 3 (i.q.r. 1–5; range 1–11) days. Most patients were treated with oral antibiotics alone (53 patients, 67 per cent). Fourteen patients (17 per cent) underwent revisional surgery. Twelve patients were readmitted for intravenous antibiotics (15 per cent), and most (8 patients, 67 per cent) were subsequently discharged with oral antibiotics.

### Cost of surgical site infection

The weighted cost of unplanned admission for SSI was £996.76 for an admission of 2 days or shorter, and £3926.16 for a longer admission (see *Table S2*). Six patients with SSI had an inpatient stay of 2 days or shorter, and seven had a stay of 3 days or longer. Length of stay data were missing for two patients, which were excluded from these analyses. The remaining 65 patients had their SSI treated without an inpatient stay. The estimated cost per SSI therefore was £456.94.

In the sensitivity analysis, the mean additional length of stay for all patients with SSI (0.63 days) was multiplied by the weighted average cost of additional inpatient days (£341.14) (see *Table S3*). This gives a best-case-scenario cost per SSI of £214.31.

### Microbiology of surgical site infection

In 23 patients, samples were taken for microbiological analysis. The most prevalent pathogen in patients with SSI was *Staphylococcus aureus* (11 patients, 47.8 per cent). Various other microbes were also isolated, including different species of *Streptococcus* (three patients), *Staphylococcus* (four patients), *Pseudomonas* (one patient), *Proteus* (one patient), and *Enterobacter* (one patient). Three patients had no growth on cultures and one patient had ‘skin flora’ isolated.

### Antiseptic adverse events

No serious adverse events were reported; specifically, there were no ignition fires or chemical burns beneath tourniquets. Four patients (0.2 per cent) reportedly developed a skin ‘rash’ after being exposed to alcoholic PVI (two patients) and alcoholic CHX (two patients).

## Discussion

This international multicentre, prospective cohort study supports the findings of meta-analyses on clean^12^ and contaminated^13^^,^^14^ surgery, and provides evidence to underpin international guidelines^9–11^ which advocate alcoholic CHX skin antisepsis. This study suggests that upper limb surgeons should adhere to guidance and use an alcoholic antiseptic, ideally CHX, for elective upper limb surgery. For emergency upper limb surgery, the findings are not in keeping with international guidance or the wider literature. Equally, there are still unresolved concerns around safety of alcoholic CHX in the presence of open wounds in the upper limb. Therefore, further research is necessary to determine the most safe and effective antiseptic in emergency upper limb surgery.

Due to the scale of hand surgery performed worldwide, even a small reduction in risk of SSI at the population level is likely to translate to considerable benefits for patients and healthcare services alike. Although the evidence remains unclear for emergency upper limb surgery, this study demonstrates that swapping to preoperative alcoholic CHX skin antisepsis for elective upper limb surgery may be associated with a 70 per cent reduction in risk of SSI, with no additional risk. For example, the NHS commissioned over 123 301 operations for four common hand conditions (Dupuytren’s disease, trigger finger, carpal tunnel syndrome, and cubital tunnel syndrome) during 2020^24^. If surgeons who perform surgery for these four common conditions were to change their practice and use alcoholic CHX (instead of their usual antiseptic), then based on these estimates, approximately 1484 infections might be prevented every year, translating to savings of £677 642 (USD 932 917) per annum for the NHS. Clearly the actual cost savings to health services might be considerably higher, given that: the methods used to estimate costs in this study are likely to underrepresent the true costs of SSI and the scope for direct and indirect savings (for example, from lower antibiotic usage, fewer healthcare visits, less contact time, etc.) is likely to be considerably greater; the breadth of elective upper limb surgery is greater than these four operations, but the benefits are transferable; and some clinicians used CHX-based antiseptics with a relatively low concentration of the active ingredient (for example, 0.05 per cent or 1 per cent CHX), which reduces its bactericidal potential^43^. Therefore, we see no reason for surgeons performing elective upper limb surgery to diverge from international guidance which endorses alcoholic CHX for preoperative skin antisepsis.

This study provides data to challenge dogma in several areas of upper limb surgery. First, the provision of preoperative antibiotics (either as a short oral course in the days leading up to emergency surgery or as a one-off dose at the time of anaesthesia induction) was not associated with a reduced risk of SSI in either the elective or emergency surgery models. This is in keeping with the wider literature on perioperative antibiotic use in upper limb surgery^34^^,^^35^^,^^44^^,^^45^ which demonstrates no benefit from (pre- or postoperative) antibiotics in patients who have no clinical features of infection and who are destined for surgery. As we approach an existential crisis surrounding antimicrobial resistance^25^, surgeons could consider exercising greater restraint and prescribe fewer antibiotics until definitive evidence from high-quality multicentre randomised trials is generated. Second, the method of wound closure was not associated with risk of infection, which is in agreement with the evidence^33^^,^^36^^,^^46^; moreover, when absorbable sutures are coated with an antimicrobial (Triclosan), then the reduced SSI risk is associated with considerable cost savings^47^. In keeping with recent work, delay from upper limb injury to surgery was not associated with the risk of SSI^33^. In this cohort, the median time to surgery for patients with open (contaminated or dirty) wounds was 27 hours and the distribution of time had a considerable positive skew (90th percentile 4 days, 95th percentile 7 days, and 99th percentile 19 days), meaning that data were captured from patients with relatively extreme delays to surgery and such delays (after various adjustments) were not associated with an increased risk of SSI.

There were no serious adverse events related to antiseptic use in this study, which is in keeping with the literature^48^. A network meta-analysis of antiseptics in 14 953 patients undergoing clean surgery found no reports of ignition fires or burns beneath tourniquets and synthesized a pooled prevalence of contact dermatitis of 1 per cent, which only occurred in patients exposed to PVI^12^. Two other systematic reviews demonstrated that skin reactions are equally rare for PVI and CHX antiseptics^49^ and chemical burns beneath tourniquets also occur with aqueous PVI^50^. There were no alcohol ignition fires, which is also in keeping with the literature. Overall, this study adds to the evidence to suggest that alcoholic CHX is safe in tourniquet-controlled upper limb surgery, and with increasing use of wide-awake local anaesthesia with no tourniquet (WALANT) surgery, this may become a moot point.

Missing data are ubiquitous in clinical research. Although reasonable steps were taken to acquire missing data (by contacting collaborators), a small proportion of required data was still missing and consequently, the final model might not be representative, standard errors may be inflated, and potentially valuable data might have been discarded. Equally, there may be confounding factors which were not captured, and heterogeneity of patients, antiseptics (see *Table S1*), operations performed, and local practices (see *Table S8*) could explain the variability in SSI following emergency surgery (for example, severity of trauma, types of wound contamination, degree of debridement).

Most infections after elective surgery occurred within 15 postoperative days. However, infections in patients undergoing elective surgery who received alcoholic CHX (*Fig. 1*) occurred with a delay of several weeks. Small amounts of CHX are known to penetrate the stratum corneum and exert bactericidal activity for hours (and potentially days) after application, an effect which may be potentiated by lipid disruption from the alcoholic solvent^51^. It is speculated that delayed infections in the alcoholic CHX were due to deep (for example, implant) infections, and future iterations of the CIPHUR portfolio of work will capture information on the exact operation performed and whether foreign materials were left, to better understand this topic.

This study cannot address the pervasive belief that topical alcohol is hazardous in the presence of open wounds. Surgical teaching is that alcohol damages tissue, and thus impairs healing, and is toxic to vital structures which may be exposed in wounds (such as peripheral nerves), and it should therefore be avoided. The limited *in*  *vitro* evidence to date^52^ does not substantiate the dogma. However, there is still insufficient literature to draw reliable conclusions about the best antiseptic in the presence of open wounds in the upper limb.

The findings of this study align with the global evidence base and international guidance regarding clean surgery, suggesting that alcoholic CHX should be used for skin antisepsis before clean (elective upper limb) surgery. For emergency (contaminated or dirty) upper limb surgery, the findings of this study were unclear and contradict the available evidence, suggesting that further research is necessary.

## Collaborators

R. Bindra (Gold Coast University Hospital, Southport, Australia), M. Sher (Gold Coast University Hospital, Southport, Australia), M. Thomas (Gold Coast University Hospital, Southport, Australia), S. D. J. Morgan (Gold Coast University Hospital, Southport, Australia), B. Hwang (Sydney Hospital, Sydney, Australia), W. Santucci (Footscray Hospital, Melbourne, Australia), P. Tran (Footscray Hospital, Melbourne, Australia), L. Kopp (Masaryk Hospital, Krajska Zdravotni, U. n. Labem, Czech Republic), V. Kunc (Masaryk Hospital, Krajska Zdravotni, Usti nad Labem, Czech Republic), A. Hamdi (Al-Azhar University Hospitals, Egypt), P. P. Grieve (Blackrock Clinic, Dublin, Ireland), S. A. Mukhaizeem (St Vincent’s University Hospital, Dublin, Ireland), K. Blake (St Vincent’s University Hospital, Dublin, Ireland), C. Cuggy (St Vincent’s University Hospital, Dublin, Ireland), R. Dolan (St Vincent’s University Hospital, Dublin, Ireland), E. Downes (St Vincent’s University Hospital, Dublin, Ireland), E. Geary (St Vincent’s University Hospital, Dublin, Ireland), A. Ghadge (St Vincent’s University Hospital, Dublin, Ireland), P. Gorman (St Vincent’s University Hospital, Dublin, Ireland), M. Jonson (St Vincent’s University Hospital, Dublin, Ireland), N. Jumper (St Vincent’s University Hospital, Dublin, Ireland), S. Kelly (St Vincent’s University Hospital, Dublin, Ireland), L. Leddy (St Vincent’s University Hospital, Dublin, Ireland), M. E. McMahon (St Vincent’s University Hospital, Dublin, Ireland), C. McNamee (St Vincent’s University Hospital, Dublin, Ireland), P. Miller (St Vincent’s University Hospital, Dublin, Ireland), B. Murphy (St Vincent’s University Hospital, Dublin, Ireland), L. O'Halloran (St Vincent’s University Hospital, Dublin, Ireland), K. O’Shea (St Vincent’s University Hospital, Dublin, Ireland), J. Skeens (St Vincent’s University Hospital, Dublin, Ireland), S. Staunton (St Vincent’s University Hospital, Dublin, Ireland), F. Timon (St Vincent’s University Hospital, Dublin, Ireland), J. Woods (St Vincent’s University Hospital, Dublin, Ireland), U. Cortinovis (IRCCS Fondazione Istituto Nazionale Tumori, Milan, Italy), L. Sala (IRCCS Fondazione Istituto Nazionale Tumori, Milan, Italy), V. Zingarello (IRCCS Fondazione Istituto Nazionale Tumori, Milan, Italy), M. H. Jusoh (Hospital Universiti Sains Malaysia, Malaysia), A. N. Sadagatullah (Hospital Universiti Sains Malaysia, Malaysia), G. Georgieva (University Clinic for Plastic and Reconstructive Surgery, Skopje, North Macedonia), S. Pejkova (University Clinic for Plastic and Reconstructive Surgery, Skopje, North Macedonia), B. Nikolovska (University Clinic for Plastic and Reconstructive Surgery, Skopje, North Macedonia), B. Srbov (University Clinic for Plastic and Reconstructive Surgery, Skopje, North Macedonia), H. K. S. Hamid (East Nile Hospital, Sudan), M. Mustafa (East Nile Hospital, Sudan), M. Abdelrahman (Soba University Hospital, Khartoum, Sudan), S. M. M. Amin (Soba University Hospital, Khartoum, Sudan), D. Bhatti (Aberdeen Royal Infirmary, Aberdeen, UK), K. M. A. Rahman (Aberdeen Royal Infirmary, Aberdeen, UK), I. Jumabhoy (Bradford Royal Infirmary, Bradford, UK), J. Kiely (Bradford Royal Infirmary, Bradford, UK), I. Kieran (Bradford Royal Infirmary, Bradford, UK), A. C. Q. Lo (Cambridge University Hospitals NHS Foundation Trust, UK), K. Y. Wong (Cambridge University Hospitals NHS Foundation Trust, UK), A. Y. Allan (Chelsea and Westminster Hospitals, London, UK), H. Armes (Chelsea and Westminster Hospitals, London, UK), M. D. Horwitz (Chelsea and Westminster Hospitals, London, UK), L. Ioannidi (Chelsea and Westminster Hospitals, London, UK), G. Masterton (Chelsea and Westminster Hospitals, London, UK), H. Chu (Derriford Hospital, Plymouth, UK), G. D. Talawadekar (Furness General Hospital, Barrow-in-Furness, UK), K. S. Tong (Furness General Hospital, Barrow-in-Furness, UK), M. Chan (Gloucestershire Hospitals NHS foundation trust, UK), M. Tredgett (Gloucestershire Hospitals NHS foundation trust, UK), C. Hardie (Harrogate District Hospital, Harrogate, UK), E. Powell-Smith (Harrogate District Hospital, Harrogate, UK), N. Gilham (Horton General Hospital, Banbury, UK), M. Prokopenko (Horton General Hospital, Banbury, UK), R. Ahmad (Huddersfield Royal Infirmary, Huddersfield, UK), J. Davies (Huddersfield Royal Infirmary, Huddersfield, UK), S. Zhen (Huddersfield Royal Infirmary, Huddersfield, UK), D. Dargan (Hull University Teaching Hospitals NHS Trust, Kingston-Upon-Hull, UK), R. M. Pinder (Hull University Teaching Hospitals NHS Trust, Kingston-Upon-Hull, UK), M. Koziara (James Cook University Hospital, Middlesbrough, UK), R. Martin (James Cook University Hospital, Middlesbrough, UK), E. Reay (James Cook University Hospital, Middlesbrough, UK), E. Cochrane (Leeds Teaching Hospitals Trust, Leeds, UK), A. Elbatawy (Leeds Teaching Hospitals Trust, Leeds, UK), F. Green (Leeds Teaching Hospitals Trust, Leeds, UK), T. Griffiths (Leeds Teaching Hospitals Trust, Leeds, UK), G. Higginbotham (Leeds Teaching Hospitals Trust, Leeds, UK), S. Louette (Leeds Teaching Hospitals Trust, Leeds, UK), G. McCauley (Leeds Teaching Hospitals Trust, Leeds, UK), I. Natalwala (Leeds Teaching Hospitals Trust, Leeds, UK), E. Salt (Leeds Teaching Hospitals Trust, Leeds, UK), R. Ahmed (Lister Hospital, Stevenage, UK), P. Goon (Lister Hospital, Stevenage, UK), R. Manton (Lister Hospital, Stevenage, UK), N. Segaren (Lister Hospital, Stevenage, UK), G. Cheung (Liverpool University Foundation Trust, Liverpool, UK), R. Mahoney (Morriston Hospital, Swansea, UK), S. Sen (Noble's Hospital, Isle of Man, UK), D. Clarkson (Nottingham City Hospital, Nottingham, UK), M. Collins (Nottingham City Hospital, Nottingham, UK), A. Bolt (Nuffield Orthopaedic Centre, Oxford, UK), P. Lokanathan (Pinderfields General Hospital, Wakefield, UK), A. Ng (Pinderfields General Hospital, Wakefield, UK), G. Jones (Peterborough City Hospital, Peterborough, UK), J. W. M. Jones (Peterborough City Hospital, Peterborough, UK), R. Kabariti (Princess Royal Hospital, Telford, UK), S. J. Rhee (Princess Royal Hospital, Telford, UK), J. Herron (Queen Elizabeth Hospital, Birmingham, UK), A. Kay (Queen Elizabeth Hospital, Birmingham, UK), L. K. Cheung (Queen Victoria Hospital, East Grinstead, UK), D. Thomson (Queen Victoria Hospital, East Grinstead, UK), R. S. Jugdey (Royal Blackburn Hospital, UK), H. Yoon (Royal Blackburn Hospital, UK), Z. L. (Royal Bournemouth Hospital, UK), J. Southgate (Royal Bournemouth Hospital, UK), C. Brennan (Royal Cornwall Hospital, Truro, UK), S. Kiani (Royal Cornwall Hospital, Truro, UK), M. Zabaglo (Royal Cornwall Hospital, Truro, UK), Z. A. Haider (Royal Cornwall Hospital, Truro, UK), R. Poulter (Royal Cornwall Hospital, Truro, UK), A. Sheik-Ali (Royal Devon & Exeter Hospital, UK), A. Watts (Royal Devon & Exeter Hospital, UK), B. Jemec (Royal Free Hospital, London, UK), N. Redgrave (Royal Free Hospital, London, UK), L. Dupley (Royal Preston Hospital, Preston, UK), M. Greenhalgh (Royal Preston Hospital, Preston, UK), J. Vella (Royal Preston Hospital, Preston, UK), H. Harris (Royal Sussex County Hospital, Sussex, UK), A. V. Robinson (Royal Sussex County Hospital, Sussex, UK), S. Dupre (Royal Victoria Infirmary, Newcastle, UK), S. Teelucksingh (Royal Victoria Infirmary, Newcastle, UK), A. Gargan (St Mary's Hospital, Paddington, London, UK), S. Hettiaratchy (St Mary's Hospital, Paddington, London, UK), A. Jain (St Mary's Hospital, Paddington, London, UK), R. Kwasnicki (St Mary's Hospital, Paddington, London, UK), A. Lee (St Mary's Hospital, Paddington, London, UK), M. Thakkar (St Mary's Hospital, Paddington, London, UK), D. Berwick (Stoke Mandeville Hospital, Aylesbury, UK), N. Ismail (Stoke Mandeville Hospital, Aylesbury, UK), M. Mahdi (Stoke Mandeville Hospital, Aylesbury, UK), J. Rodrigues (Stoke Mandeville Hospital, Aylesbury, UK), C. Liew (University College Hospital, London, UK), A. Saadya (University College Hospital, London, UK), M. Clarkson (University Hospital Wishaw, Wishaw, UK), C. Brady (Wexham Park Hospital, Slough, UK), R. Harrison (Wexham Park Hospital, Slough, UK), A. Rayner (Wexham Park Hospital, Slough, UK), G. Nolan (Whiston hospital, Liverpool, UK), B. Phillips (Whiston hospital, Liverpool, UK), N. Madhusudan (Wirral University Teaching Hospital, Birkenhead, UK).

## Funding

R.G.W. is a Doctoral Research Fellow funded by the National Institute for Health Research (NIHR) (DRF-2018–11-ST2-028). J.P.T. is a Clinical Lecturer funded by Health Education England (HEE)/NIHR. J.C.R.W. is a Research Fellow funded by the Royal College of Surgeons of England and the British Society of Surgery for the Hand. The views expressed in this publication are those of the author(s) and not necessarily those of the NIHR, NHS, or the UK Department of Health and Social Care.

## Supplementary Material

zrab117_Supplementary_Data
