# Supplementary material for: Chlorhexidine versus povidone–iodine skin antisepsis before upper limb surgery (CIPHUR): an international multicentre prospective cohort study
Source: BJS Open. 2021 Dec 15;5(6):zrab117. doi: 10.1093/bjsopen/zrab117 (PMC8677347; doi:10.1093/bjsopen/zrab117)
Supplement: zrab117_Supplementary_Data [file zrab117_supplementary_data.zip › Appendix_1.docx]

**Appendix 1: The CIPHUR Collaborative**

Authors are listed alphabetically by country, hospital and surname

| **Country** | **Hospital, City** | **Co-author(s)** |
| --- | --- | --- |
| **Australia** | Gold Coast University Hospital, Southport | Randy Bindra, Mustafa Sher, Michael Thomas, Samuel DJ Morgan |
|  | Sydney Hospital, Sydney | Bridget Hwang |
|  | Footscray Hospital, Melbourne | Walter Santucci, Phong Tran |
| **Czech Republic** | Masaryk Hospital, Krajska Zdravotni, Usti nad Labem | Lubomir Kopp, Vojtěch Kunc |
| **Egypt** | Al-Azhar University Hospitals | Abdelnaser Hamdi |
| **Ireland** | Blackrock Clinic, Dublin | Philip P Grieve |
|  | St Vincent’s University Hospital, Dublin | Sammy Al Mukhaizeem, Kealan Blake, Conor Cuggy, Róisín Dolan, Eoin Downes, Ellen Geary, Anooj Ghadge, Patrick Gorman, Miles Jonson, Natalie Jumper, Stephen Kelly, Lauren Leddy, Mary Ellen McMahon, Conor McNamee, Peggy Miller, Ben Murphy, Louis O'Halloran, Kieran O’Shea, Joshua Skeens, Susannah Staunton, Freya Timon, Jack Woods, |
| **Italy** | IRCCS Fondazione Istituto Nazionale Tumori, Milan | Umberto Cortinovis, Laura Sala, Valeria Zingarello |
| **Malaysia** | Hospital Universiti Sains Malaysia | Mohd Hanifah Jusoh, Abdul Nawfar Sadagatullah |
| **North Macedonia** | University Clinic for Plastic and Reconstructive Surgery, Skopje | Gordana Georgieva, Sofija Pejkova, Bisera Nikolovska, Blagoja Srbov |
| **Sudan** | East Nile Hospital | Hytham K S Hamid, Muaaz Mustafa |
|  | Soba University Hospital, Khartoum | Mohamed Abdelrahman, Sara M M Amin |
| **United Kingdom** | Aberdeen Royal Infirmary | Dujanah Bhatti, Kaz M A Rahman |
|  | Bradford Royal Infirmary, Bradford | Irfan Jumabhoy, John Kiely, Ingrid Kieran |
|  | Cambridge University Hospitals NHS Foundation Trust | Andre C Q Lo, Kai Yuen Wong |
|  | Chelsea and Westminster Hospitals, London | Anna Y Allan, Henry Armes, Maxim D Horwitz, Lydia Ioannidi, Gary Masterton |
|  | Derriford Hospital, Plymouth | Howard Chu |
|  | Furness General Hospital, Barrow-in-Furness | Gautam Deepak Talawadekar, Kin Seng Tong |
|  | Gloucestershire Hospitals NHS foundation trust | Matthew Chan, Michael Tredgett |
|  | Harrogate District Hospital, Harrogate | Claire Hardie, Edward Powell-Smith |
|  | Horton General Hospital, Banbury | Nicolas Gilham, Max Prokopenko |
|  | Huddersfield Royal Infirmary, Huddersfield | Reddin Ahmad, James Davies, Shuyi  Zhen |
|  | Hull University Teaching Hospitals NHS Trust, Kingston-Upon-Hull | Dallan Dargan, Richard M Pinder, Joshua P Totty |
|  | James Cook University Hospital, Middlesborough | Michal Koziara, Rebecca Martin, Emma Reay |
|  | Leeds Teaching Hospitals Trust, Leeds | Elliott Cochrane, Amr Elbatawy, Frederick Green, Timothy Griffiths, George Higginbotham, Stefan Louette, Gordon McCauley, Ibrahim Natalwala, Emily Salt |
|  | Lister Hospital, Stevenage | Rowaa Ahmed, Patrick Goon, Robert Manton, Nicholas Segaren |
|  | Liverpool University Foundation Trust, Liverpool | Graham Cheung |
|  | Morriston Hospital, Swansea | Rachel Mahoney |
|  | Noble's Hospital, Isle of Man | Suddhajit Sen |
|  | Nottingham City Hospital, Nottingham | David Clarkson, Michelle Collins |
|  | Nuffield Orthopaedic Centre, Oxford | Alexander Bolt |
|  | Pinderfields General Hospital, Wakefield | Prashan Lokanathan, Aaron Ng |
|  | Peterborough City Hospital, Peterborough | Georgina Jones, Jonathan W M Jones |
|  | Princess Royal Hospital, Telford | Rakan Kabariti, Shin Jae Rhee |
|  | Queen Elizabeth Hospital, Birmingham | Jonathan Herron, Alan Kay |
|  | Queen Victoria Hospital, East Grinstead | Lok Ka Cheung, David Thomson |
|  | Royal Blackburn Hospital | Ravi Singh Jugdey, Heewon Yoon |
|  | Royal Bournemouth Hospital | Zoe Lin, Jeremy Southgate |
|  | Royal Cornwall Hospital, Truro | Ciaran Brennan, Shahavez Kiani, Mate Zabaglo, Zulfiqar Ali Haider, Rob Poulter |
|  | Royal Devon & Exeter Hospital | Azizi Sheik-Ali, Andrew Watts |
|  | Royal Free Hospital, London | Barbara Jemec, Natalie Redgrave |
|  | Royal Preston Hospital, Preston | Leanne Dupley, Michael Greenhalgh, John Vella |
|  | Royal Sussex County Hospital, Sussex | Holly Harris, Alyss V Robinson |
|  | Royal Victoria Infirmary, Newcastle | Sophie Dupre, Sachin Teelucksingh |
|  | St Mary's Hospital, Paddington, London | Alice Gargan, Shehan Hettiaratchy, Abhilash Jain, Richard Kwasnicki, Alice Lee, Mehul Thakkar |
|  | Stoke Mandeville Hospital, Aylesbury | Daniele Berwick, Nizar Ismail, Muntazir Mahdi,  Jeremy Rodrigues |
|  | University College Hospital, London | Colin Liew, Ahmad Saadya |
|  | University Hospital Wishaw, Wishaw | Michelle Clarkson |
|  | Wexham Park Hospital, UK | Chevonne Brady, Rachael Harrison, Anthony Rayner |
|  | Whiston hospital, Liverpool | Grant Nolan, Ben Phillips |
|  | Wirral University Teaching Hospital, Birkenhead | Namrata Madhusudan |
