# Supplementary material for: Chlorhexidine versus povidone–iodine skin antisepsis before upper limb surgery (CIPHUR): an international multicentre prospective cohort study
Source: BJS Open. 2021 Dec 15;5(6):zrab117. doi: 10.1093/bjsopen/zrab117 (PMC8677347; doi:10.1093/bjsopen/zrab117)
Supplement: zrab117_Supplementary_Data [file zrab117_supplementary_data.zip › Supplementary_material.docx]

**Table of contents**

**Figure S1.** A bar chart showing when patients were recruited to CIPHUR. There was a substantial fall in recruitment during the 1^st^ wave of the COVID-19 pandemic in March-April 2020.

**Figure S2.** A map showing the hospitals included in the CIPHUR

**Figure S3.** Forest plot of risk ratios for surgical site infection derived from mixed-effects logistic regression

**Table S1.** Composition of antiseptics and how they were grouped

**Table S2.** Weighted average of two HRG codes related to unplanned admissions for surgical site infections

**Table S3.** Weighted average of two HRG codes related to excess bed days for surgical site infections

**Table S4.** Characteristics of patients undergoing emergency upper limb surgery

**Table S5.** Characteristics of patients undergoing elective surgery

**Table S6.** Risk of Surgical Site Infection derived from mixed-effects logistic regression. The location (i.e., the hospital,) was associated with considerable variability in the risk of SSI in emergency model (ICC 0.17 [95% CI 0.04, 0.50]) but this was explained by one outlier unit and when this hospital was removed in a sensitivity analysis, the residual variance was effectively zero (ICC 1.49x10^-33^ [95% CI 8.74x10^-34^, 2.54x10^-33^]). Location did not affect the risk of SSI in the elective surgery model (ICC 6.85x10^-34^ [95% CI 2.79x10^-34^, 1.68x10^-33^]).

**Table S7.** Predicted benefits if surgeons changed their practice and used alcoholic CHX, based data 123,301 operations performed in the UK for dupuytren’s disease, trigger finger release, cubital tunnel and carpal tunnel decompression during 2020.

**Table S8.** Recruitment by hospital, city and country

**Figure S1.** A bar chart showing when patients were recruited to CIPHUR. There was a substantial fall in recruitment during the 1^st^ wave of the COVID-19 pandemic in March-April 2020.

**Figure S2.** A map showing the hospitals included in the CIPHUR


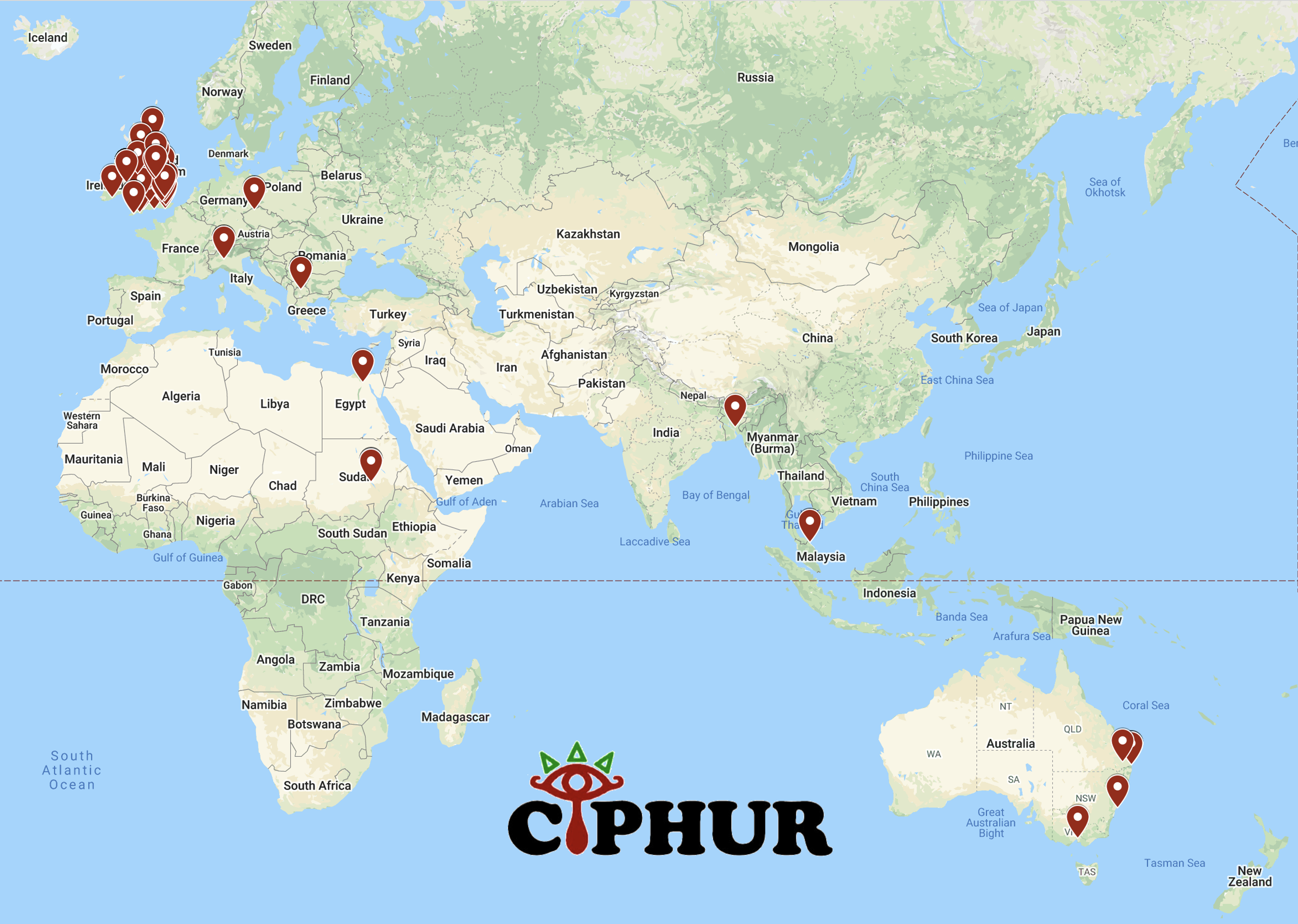


**Figure S3.** Forest plot of risk ratios for surgical site infection derived from mixed-effects logistic regression

**Table S1.** Composition of antiseptics and how they were grouped

| **Antiseptic grouping** | **Antiseptic Used** | **Frequency (%)** |
| --- | --- | --- |
| Aqueous Povidone-Iodine | Aqueous povidone-iodine 1% | 42 |
|  | Aqueous povidone-iodine 7.5% | 163 |
|  | Aqueous povidone-iodine 10% | 435 |
|  | Unspecified concentration | 3 |
| Alcoholic povidone-iodine | Alcoholic povidone-iodine 1% | 37 |
|  | Alcoholic povidone-iodine 7.5% | 32 |
|  | Alcoholic povidone-iodine 10% | 226 |
|  | Unspecified concentration | 12 |
| Aqueous chlorhexidine | Aqueous chlorhexidine 0.05% | 208 |
|  | Aqueous chlorhexidine 0.5% | 186 |
|  | Aqueous chlorhexidine 2% | 61 |
|  | Aqueous chlorhexidine 4% | 35 |
|  | Unspecified concentration | 49 |
| Alcoholic chlorhexidine | Alcoholic chlorhexidine 0.5% | 236 |
|  | Alcoholic chlorhexidine 2% | 501 |
|  | Alcoholic chlorhexidine 4% | 50 |
|  | Unspecified concentration | 17 |
| Other | SoftSept N (ethanol 74.1% and 2-propanol 10%) | 28 |
|  | Kodan tincture (2-propanol 45%, 1-propanol 10%, biphenyl-2-ol 0.2%, H_2_O_2_ 30%, water) | 14 |
|  | Chlorhexidine Acetate 0.015% & Cetrimide 0.15% in water | 13 |
|  | Tisept (Chlorhexidine Gluconate 0.015% & Cetrimide 0.15% in water) | 10 |
|  | None | 5 |
|  | Alcoholic chlorhexidine 2% and alcoholic povidone-iodine 10% | 4 |
|  | Ethanol 74.1%, 2-propanol 10%, water 15.9% | 2 |
|  | 70% alcohol (unspecified) | 1 |
|  | Alcoholic povidone-iodine 10% and alcoholic chlorhexidine 2% | 1 |
|  | Chlorhexidine gluconate 0.05% and Cetrimide 0.5% in water | 1 |

**Table S2.** Weighted average of two HRG codes related to unplanned admissions for surgical site infections

| **HRG Code** | **Description** | **Short Stay** | | **Long Stay** | |
| --- | --- | --- | --- | --- | --- |
|  |  | **Activity** | **Unit Cost** | **Activity** | **Unit Cost** |
| WH07C | Infections or Other Complications of Procedures, with Single Intervention, with CC Score 2+ | 70 | £856 | 1,521 | £5,063 |
| WH07D | Infections or Other Complications of Procedures, with Single Intervention, with CC Score 0-1 | 392 | £997 | 3,572 | £3,321 |
|  | **Weighted Average (Inflation adjusted)** | **£996.76** | | **£3926.16** | |

**Table S3.** Weighted average of two HRG codes related to excess bed days for surgical site infections

| **HRG Code** | **Description** | **Activity** | **Unit Cost** |
| --- | --- | --- | --- |
| WH07C | Infections or Other Complications of Procedures, with Single Intervention, with CC Score 2+ | 1463 | £332 |
| WH07D | Infections or Other Complications of Procedures, with Single Intervention, with CC Score 0-1 | 2888 | £324 |
|  | **Weighted Average (Inflation Adjusted)** | **£341.14** | |

**Table S4.** Characteristics of patients undergoing emergency upper limb surgery

| **Characteristics** | | **No SSI (n=1365)** | **SSI (n=57)** | **p-value** |
| --- | --- | --- | --- | --- |
| Wound toilet (%) | None | 288 (21) | 18 (32) | 0·070 |
|  | Tap water | 28 (2) | 5 (8) | 0·009 |
|  | Sterile water or saline | 453 (33) | 16 (28) | 0·474 |
|  | Povidone-Iodine | 113 (8) | 4 (7) | 1·000 |
|  | Chlorhexidine | 34 (3) | 2 (4) | 0·653 |
| Antibiotics between assessment & surgery (%) | | 573 (42) | 32 (56) | 0·055 |
| Median hours form injury to surgery (IQR) | | 39 (20, 87) | 44 (19) | 0·119 |
| Type of anaesthesia (%) | Local | 450 (95) | 23 (5) | 0·442 |
|  | Regional | 182 (97) | 5 (3) |  |
|  | General | 733 (96) | 29 (4) |  |
| Antibiotics at induction of anaesthesia (%) | | 732 (54) | 757 (53) | 0·175 |
| Grade of operating surgeon (%) | Junior trainee | 82 (98) | 2 (2) | 0·682 |
|  | Specialty trainee | 634 (96) | 25 (4) |  |
|  | Consultant | 649 (96) | 30 (4) |  |
| Tourniquet (%) | | 941 (69) | 36 (63) | 0·382 |
| Bilateral surgery (%) | | 14 (1) | 4 (7) | 0·005 |
| Informal (“social”) wash before skin preparation (%) | | 272 (21) | 17 (32) | 0·087 |
| Antiseptic (%) | Aqueous PVI | 357 (93) | 25 (7) | 0·016 |
|  | Alcoholic PVI | 206 (99) | 2 (1) |  |
|  | Aqueous CHX | 383 (96) | 14 (4) |  |
|  | Alcoholic CHX | 401 (96) | 16 (4) |  |
|  | Others | 14 (100) | 0 (0) |  |
| Who applied the antiseptic (%) | Theatre staff | 423 (96) | 18 (4) | 1·000 |
|  | Surgeon | 914 (96) | 38 (4) |  |
| How was the antiseptic applied (%) | Single use applicator | 65 (93) | 5 (7) | 0·201 |
|  | Decanted solution applied using a swab/sponge held in an instrument | 1282 (96) | 52 (4) |  |
| Method of wound closure (%) | Absorbable sutures | 691 (96) | 31 (4) | 0·586 |
|  | Non-absorbable sutures | 548 (96) | 24 (4) |  |
|  | Combination of absorbable and non-absorbable sutures | 43 (100) | 0 (0) |  |
|  | Healing by secondary intention | 34 (94) | 2 (6) |  |
|  | No wound(s) to close | 30 (100) | 0 (0) |  |
| Median total operative time in minutes (IQR) | | 51 (30, 75) | 55 (33, 98) | 0·182 |
| Postoperative antibiotics | | 795 (58) | 40 (70) | 0·076 |

**Table S5.** Characteristics of patients undergoing elective surgery

| **Characteristics** | | **No SSI (n=973)** | **SSI (n=28)** | **p-value** |
| --- | --- | --- | --- | --- |
| Preoperative antibiotics (%) | | 75 (8) | 1 (4) | 0·717 |
| Type of anaesthesia (%) | Local | 410 (98) | 10 (2) | 0·778 |
|  | Regional | 136 (97) | 4 (3) |  |
|  | General | 427 (97) | 14 (3) |  |
| Antibiotics at induction of anaesthesia (%) | | 354 (36) | 11 (39) | 0·843 |
| Grade of operating surgeon (%) | Junior trainee | 17 (94) | 1 (6) | 0·278 |
|  | Specialty trainee | 191 (98) | 3 (2) |  |
|  | Consultant | 765 (97) | 24 (3) |  |
| Tourniquet (%) | | 608 (62) | 15 (53) | 0·332 |
| Bilateral surgery (%) | | 21 (2) | 3 (11) | 0·027 |
| Antiseptic scrub before formal skin prep (%) | | 98 (11) | 0 (0) | 0·100 |
| Antiseptic (%) | Aqueous PVI | 245 (96) | 9 (4) | 0·225 |
|  | Alcoholic PVI | 93 (97) | 3 (3) |  |
|  | Aqueous CHX | 199 (97) | 6 (3) |  |
|  | Alcoholic CHX | 371 (98) | 6 (2) |  |
|  | Other | 65 (94) | 4 (6) |  |
| Who applied the antiseptic (%) | Theatre staff | 392 (97) | 13 (3) | 0·428 |
|  | Surgeon | 541 (98) | 13 (2) |  |
| How was the antiseptic applied (%) | Single use applicator | 198 (98) | 4 (2) | 0·630 |
|  | Decanted solution applied using a swab/sponge held in an instrument | 750 (97) | 23 (3) |  |
| Method of wound closure (%) | Absorbable sutures | 396 (41) | 9 (32) | 0·775 |
|  | Non-absorbable sutures | 427 (44) | 15 (54) |  |
|  | Combination of absorbable and non-absorbable sutures | 118 (12) | 4 (14) |  |
|  | Healing by secondary intention | 16 (2) | 0 (0) |  |
|  | No wound(s) to close | 8 (1) | 0 (0) |  |
| Median total operative time in minutes (IQR) | | 41 (22, 70) | 45 (21, 75) | 0·957 |
| Postoperative antibiotics | | 207 (21) | 5 (18) | 0·816 |

**Table S6.** Risk of Surgical Site Infection derived from mixed-effects logistic regression. The location (i.e., the hospital,) was associated with considerable variability in the risk of SSI in emergency model (ICC 0.17 [95% CI 0.04, 0.50]) but this was explained by one outlier unit and when this hospital was removed in a sensitivity analysis, the residual variance was effectively zero (ICC 1.49x10^-33^ [95% CI 8.74x10^-34^, 2.54x10^-33^]). Location did not affect the risk of SSI in the elective surgery model (ICC 6.85x10^-34^ [95% CI 2.79x10^-34^, 1.68x10^-33^]).

| **Risk Factors** | **Unadjusted RR (95% CI)** | **Adjusted RR (95% CI)** |
| --- | --- | --- |
| **Emergency surgery** |  |  |
| Aqueous Povidone-Iodine | referent | referent |
| Alcoholic Povidone-Iodine | 0.12 (0.02, 0.75) | 0.12 (0.02, 0.85) |
| Aqueous Chlorhexidine | 0.41 (0.18, 0.93) | 0.39 (0.16, 0.99) |
| Alcoholic Chlorhexidine | 0.47 (0.24, 0.93) | 0.49 (0.23, 1.05) |
| Clean wound | referent | referent |
| Contaminated wound | 2.01 (0.88, 4.60) | 1.19 (0.44, 3.22) |
| Dirty wound | 3.60 (1.19, 10.9) | 2.17 (0.53, 8.87) |
| Diabetes mellitus | 2.00 (0.75, 5.29) | 1.91 (0.79, 4.64) |
| Current smoker | 2.07 (1.15, 3.74) | 2.07 (1.16, 3.68) |
| Wound irrigation at assessment | 0.50 (0.23, 1.09) | 0.51 (0.22, 1.18) |
| Preoperative antibiotics | 0.88 (0.47, 1.62) | 0.77 (0.37, 1.58) |
| Consultant operating | 1.30 (0.76, 2.21) | 1.68 (0.85, 3.33) |
| Other wound closure method | referent | referent |
| Wound closure with absorbable sutures | 2.90 (0.61, 13.7) | 3.03 (0.60, 15.4) |
| Closed with non-absorbable sutures | 2.75 (0.58, 13.0) | 2.19 (0.51, 9.51) |
| Postoperative antibiotics | 1.86 (0.94, 3.71) | 1.19 (0.79, 3.02) |
| **Elective surgery** |  |  |
| Aqueous Povidone-Iodine | referent | referent |
| Alcoholic Povidone-Iodine | 0.88 (0.31, 2.47) | 0.87 (0.31, 2.50) |
| Aqueous Chlorhexidine | 0.82 (0.37, 2.00) | 0.79 (0.36, 1.73) |
| Alcoholic Chlorhexidine | 0.44 (0.20, 0.97) | 0.36 (0.15, 0.89) |
| Diabetes mellitus | 0.38 (0.05, 3.00) | 0.48 (0.06, 4.06) |
| Current smoker | 0.98 (0.25, 3.78) | 1.22 (0.34, 4.43) |
| Preoperative antibiotics | 0.95 (0.45, 2.01) | 0.58 (0.28, 1.19) |
| Consultant operating | 1.63 (0.57, 4.63) | 1.87 (0.63, 5.52) |
| Other wound closure method | referent | referent |
| Wound closure with absorbable sutures | 0.87 (0.21, 3.61) | 1.09 (0.31, 3.89) |
| Closed with non-absorbable sutures | 1.38 (0.24, 7.89) | 1.36 (0.51, 3.61) |
| Postoperative antibiotics | 0.80 (0.42, 1.55) | 0.89 (0.40, 1.95) |

**Table S7.** Predicted benefits if surgeons changed their practice and used alcoholic CHX, based data 123,301 operations performed in the UK for dupuytren’s disease, trigger finger release, cubital tunnel and carpal tunnel decompression during 2020.

| **Usual practice (SSI prevalence)** | Comparator (SSI prevalence) | | Absolute risk reduction (ARR) if surgeons used Alcoholic CHX | Number Needed to Treat (NNT) | Number of patients receiving the usual practice antiseptic (%) | Number of patients expected to developed SSI (%) | Number of SSIs prevent if surgeons used alcoholic CHX instead of their usual practice |
| --- | --- | --- | --- | --- | --- | --- | --- |
| Aqueous Chlorhexidine (**2.93%**) | | Alcoholic Chlorhexidine (**1.59%**) | 1.34% | 75 | 24660.2 (**20%**) | 722.54 (**2.93%**) | 330.45 |
| Alcoholic Povidone-Iodine (**3.12%**) | | Alcoholic Chlorhexidine (**1.59%**) | 1.53% | 65 | 12330.1 (**10%**) | 384.70 (**3.12%**) | 188.65 |
| Aqueous Povidone-Iodine (**3.54%**) | | Alcoholic Chlorhexidine (**1.59%**) | 1.95% | 51 | 30825.25 (**25%**) | 1091.21 (**3.54%**) | 601.09 |
| Other antiseptics (**5.80%**) | | Alcoholic Chlorhexidine (**1.59%**) | 4.21% | 24 | 8631.07 (**7%**) | 500.60 (**5.80%**) | 363.37 |
|  | | | **Mean = 2.26%** | **Mean = 44** | **Total = 76,446.62** | **Total = 2699.06** | **Total = 1483.56** |

**Table S8.** Recruitment by hospital, city and country

| **Country** | **Hospital, City** | **Number of patients recruited** |
| --- | --- | --- |
| **Australia** | Gold Coast University Hospital, Southport | 43 |
|  | Sydney Hospital, Sydney | 20 |
|  | Footscray Hospital, Melbourne | 40 |
| **Czech Republic** | Masaryk Hospital, Krajska Zdravotni, Usti nad Labem | 33 |
| **Egypt** | Al-Azhar University Hospitals | 30 |
| **Ireland** | Blackrock Clinic, Dublin | 51 |
|  | St Vincent’s University Hospital, Dublin | 445 |
| **Italy** | IRCCS Fondazione Istituto Nazionale Tumori, Milan | 92 |
| **Malaysia** | Hospital Universiti Sains Malaysia | 25 |
| **North Macedonia** | University Clinic for Plastic and Reconstructive Surgery, Skopje | 80 |
| **Sudan** | East Nile Hospital | 23 |
|  | Soba University Hospital, Khartoum | 32 |
| **United Kingdom** | Aberdeen Royal Infirmary | 21 |
|  | Bradford Royal Infirmary, Bradford | 65 |
|  | Cambridge University Hospitals NHS Foundation Trust | 53 |
|  | Chelsea and Westminster Hospitals, London | 64 |
|  | Derriford Hospital, Plymouth | 34 |
|  | Furness General Hospital, Barrow-in-Furness | 20 |
|  | Gloucestershire Hospitals NHS foundation trust | 22 |
|  | Harrogate District Hospital, Harrogate | 20 |
|  | Horton General Hospital, Banbury | 22 |
|  | Huddersfield Royal Infirmary, Huddersfield | 70 |
|  | Hull University Teaching Hospitals NHS Trust, Kingston-Upon-Hull | 46 |
|  | James Cook University Hospital, Middlesborough | 79 |
|  | Leeds Teaching Hospitals Trust, Leeds | 170 |
|  | Lister Hospital, Stevenage | 51 |
|  | Liverpool University Foundation Trust, Liverpool | 33 |
|  | Morriston Hospital, Swansea | 20 |
|  | Noble's Hospital, Isle of Man | 25 |
|  | Nottingham City Hospital, Nottingham | 21 |
|  | Nuffield Orthopaedic Centre, Oxford | 12 |
|  | Pinderfields General Hospital, Wakefield | 20 |
|  | Peterborough City Hospital, Peterborough | 20 |
|  | Princess Royal Hospital, Telford | 25 |
|  | Queen Elizabeth Hospital, Birmingham | 20 |
|  | Queen Victoria Hospital, East Grinstead | 41 |
|  | Royal Blackburn Hospital | 20 |
|  | Royal Bournemouth Hospital | 20 |
|  | Royal Cornwall Hospital, Truro | 85 |
|  | Royal Devon & Exeter Hospital | 27 |
|  | Royal Free Hospital, London | 20 |
|  | Royal Preston Hospital, Preston | 60 |
|  | Royal Sussex County Hospital, Sussex | 11 |
|  | Royal Victoria Infirmary, Newcastle | 48 |
|  | St Mary's Hospital, Paddington, London | 80 |
|  | Stoke Mandeville Hospital, Aylesbury | 76 |
|  | University College Hospital, London | 20 |
|  | University Hospital Wishaw, Wishaw | 5 |
|  | Wexham Park Hospital, UK | 46 |
|  | Whiston hospital, Liverpool | 20 |
|  | Wirral University Teaching Hospital, Birkenhead | 28 |
